# Editorial: The Insect Central Complex—From Sensory Coding to Directing Movement

**DOI:** 10.3389/fnbeh.2018.00156

**Published:** 2018-07-30

**Authors:** Stanley Heinze, Keram Pfeiffer

**Affiliations:** ^1^Department of Biology, Lund University, Lund, Sweden; ^2^Behavioral Physiology and Sociobiology (Zoology II), Biozentrum, University of Würzburg, Würzburg, Germany

**Keywords:** central complex, action selection, sensory integration, insects, brain, motor control, neuroanatomy

Structure and function of any nervous system are intimately linked. The function of the brain and its components cannot be understood without knowing their morphological characteristics, whereas the anatomical outline of the brain only gains relevance through functional insights. This is true on many levels, from entire brains, to circuits and individual neurons. Evolution has yielded elaborate, ordered arrangements of neurons in brains as diverse as those of mammals and flies, e.g., arrays of neurons with regularly intercalating branches, multilayered brain areas, or parallel sensory pathways. Those regularly occurring motifs of anatomical arrangements are promising access points for gaining insights into the fundamental computations supported by those structures and thus can lead the way to understanding brains in general.

One such region of almost crystalline regularity is the insect central complex (CX), a midline spanning conglomerate of four brain areas that is conserved across all insects (Pfeiffer and Homberg, 2014). It consists of 16–18 vertical columns that are intersected by horizontal layers, formed by repeating arrays of columnar neurons and layer-specific tangential neurons. While many types of columnar neuron provide highly specific, cross-hemispheric connections between the individual components of the CX, the tangential neurons provide input from other brain areas. Output is carried by very few types of columnar neuron and converges in premotor command centers. While input and output to this region have been characterized in some detail, the intrinsic computations carried out by this highly complex, yet ordered entanglement of neurons are largely unknown.

The function of the CX has been tackled from many angles, revealing three main roles: motor control, sensory integration, and a range of functions that can be summarized under the term “higher functions,” such as control of sleep, attention, spatial and object memory (Pfeiffer and Homberg, 2014). All those functions lie at the heart of neural control of behavior, and action-selection based on sensory information, previous experience and internal state have been proposed as a unifying function for the CX. Given that those functions are arguably among the most fundamental tasks carried out by all brains, understanding the CX could lead to fundamental insights into essential computations that underlie how brains control behavior across animals.

This Research Topic thus examines the CX from various angles. As already mentioned, the CX is highly conserved across insects. The first paper by Thoen et al. expands this view by characterizing a CX in mantis shrimps, sophisticated marine crustaceans that are otherwise renown for their exceptional color vision. While other crustacean brains lack many features of the insect CX, the authors find almost perfect resemblance between insects and the studied mantis shrimps, leading to interesting implications about the origin of this brain area, the functional necessity of its intrinsic organization and its relation to behavior.

Second, the origin of the regular CX-neuroarchitecture as well as its complex expression patterns of neurochemical substances during embryonic development is reviewed in the paper by Boyan and Liu. Given the intricate structure-function relation in the CX-circuits, understanding how the neurochemical architecture is established at the same time as the neuroanatomical architecture, together resulting in an ordered topology of neurons with distinct projection patterns and molecular identities, is key for systematically narrowing down functional roles for each cell type and has implications for the evolutionary origin of those neuron types. The latter is crucial for cross-species comparisons, which often implicitly assume that the CX is identical across species separated by hundreds of millions of years of evolution.

The second part of the Research Topic explores the relation between the gross morphology of the CX components and behavioral characteristics of the species. As sensory processing in the context of navigation and orientation is probably the best described CX-function (e.g., Heinze and Homberg, 2007; Seelig and Jayaraman, 2015; Stone et al., 2017), both papers examine navigation behaviors. Firstly, de Vries et al. compare the volume of the CX alongside functionally related brain regions between a migratory and a non-migratory moth species. In line with the idea that the basic computations carried out by this region of the brain are crucial for all insects, no large-scale differences were found that reflect the different behavioral strategies of those insects. Differences that explain these distinct behaviors therefore have to result from differences in circuit architecture that do not lead to alterations in neuropil volumes. Differently, Grob et al. compare ants before and after their first learning walks, a characteristic behavior that enables these insects to learn the arrangements of landmarks around the nest entrance for returning to home after foraging. Exposure to a natural pattern of skylight polarization during this behavior was crucial for volume increases in the CX and in the mushroom body; changes that therefore correlate with the ability of foraging ants to navigate precisely.

As mentioned before, the CX also is involved in spatial learning (e.g., Ofstad et al., 2011). While this aspect has previously been explored exclusively in flies, Plath et al. provide first insights into possible roles of the bee CX in spatial learning of color cues. The authors find an interesting division of labor between the mushroom body and the CX by pharmacologically silencing each region during the learning assay. The CX appeared to be crucial in mediating the goal directed behavioral response to the learned stimulus, while the mushroom body carried out the actual cue association.

To gain a deep understanding of how CX-neurons are involved in guiding the mentioned behaviors, the detailed outline of the neural circuits have to be illuminated. Two papers, Held et al. and Homberg and Müller, investigate the ultrastructure of neural elements in key parts of the CX in bees and locusts, respectively. Held et al. confirm that the detailed organization of input pathways involved in compass sensing is conserved between bees and locusts, whereas Homberg and Müller identify complex local interactions within the ellipsoid body (lower division of the central body) in locusts, providing an interesting dataset for comparison to similar information in flies.

By combining all known information, both anatomical and physiological, the next two papers explore possible functional implications of circuit architecture. Fiore et al. examine the local connectome of the *Drosophila* ellipsoid body. Their models result in potential roles of this neuropil in driving goal directed behaviors. Second, Kakaria and de Bivort condense all known connections of the *Drosophila* CX into a spiking model and reveal that this is sufficient to yield an array of head direction cells in the protocerebral bridge, as was recently observed experimentally (Seelig and Jayaraman, 2015). Adding to this, Givon et al. present a novel, web-based tool that allows to visualize activity in CX models to efficiently evaluate the functional consequences of altered connections, e.g., in mutants, or to test hypotheses about CX-function *in silico*.

Finally, the review by Varga et al. covers recent insights into the function of the cockroach CX, linking sensory integration and motor control in exquisite detail. The authors further explore the resemblance of functional concepts between vertebrate brains and the insect CX, alluding to the possibility that, indeed, we can gain fundamental insights into general brain function by studying the tiny brains of insects.

## Author contributions

SH wrote the initial draft. KP reviewed and edited the manuscript.

### Conflict of interest statement

The authors declare that the research was conducted in the absence of any commercial or financial relationships that could be construed as a potential conflict of interest.
